# Supplementary material for: Preclinical Evaluation of Invariant Natural Killer T Cells Modified with CD38 or BCMA Chimeric Antigen Receptors for Multiple Myeloma
Source: Int J Mol Sci. 2021 Jan 22;22(3):1096. doi: 10.3390/ijms22031096 (PMC7865760; doi:10.3390/ijms22031096)
Supplement: Supplementary file 1 [file ijms-22-01096-s001.pdf]

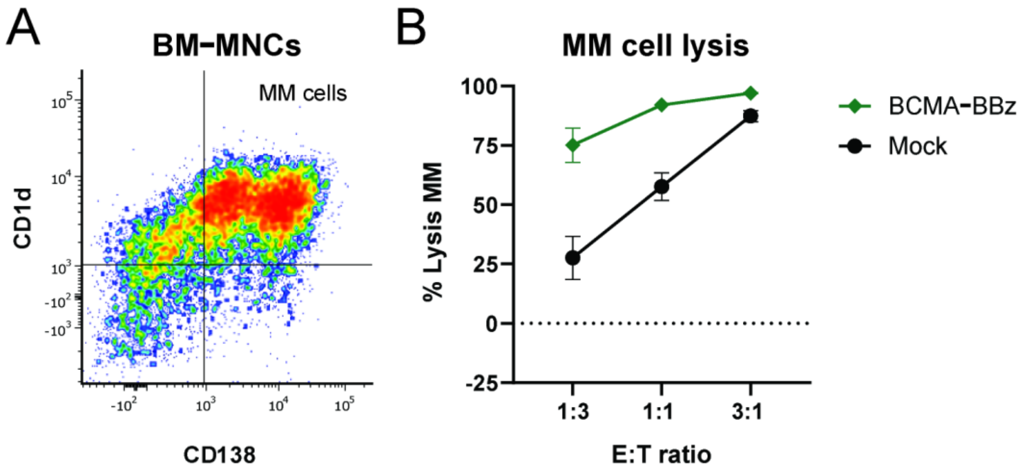

Figure S1. Cytotoxic activity of BCMA-CAR and Mock transduced iNKT cells against CD1d⁺ primary MM cells. (**a**) Density plot depicting the expression of CD1d and CD138, upper right quadrant represent the plasma cells. (**b**) Graph demonstrates lysis the of CD38⁺CD138⁺ MM cells by BCMA-BBz CAR iNKT cells or Mock transduced iNKT cells after 16 hour co-incubation. Note the high levels of lysis by Mock transduced iNKT cells at high E:T ratios, which is probably mediated via the endogenous TCR.
